# *Hydrops fetalis* caused by a complex congenital heart defect with concurrent hypoplasia of pulmonary blood vessels and lungs visualized by micro-CT in a French Bulldog

**DOI:** 10.1186/s12917-024-04060-5

**Published:** 2024-05-11

**Authors:** Olga Szaluś-Jordanow, Karolina Barszcz, Wojciech Mądry, Michał Buczyński, Michał Czopowicz, Adam Gierulski, Agata Moroz-Fik, Marcin Mickiewicz, Michał Grzegorczyk, Jakub Jaroszewicz

**Affiliations:** 1 Department of Small Animal Diseases with Clinic, Institute of Veterinary Medicine, Warsaw University of Life Sciences-SGGW, Nowoursynowska Str. 159c, Warsaw, 02-776 Poland; 2 Department of Morphological Sciences, Institute of Veterinary Medicine, Warsaw University of Life Sciences-SGGW, Nowoursynowska 159, Warsaw, 02-776 Poland; 3 Department of Heart, Chest and Transplant Surgery, Medical University of Warsaw, Żwirki i Wigury 63A, Warsaw, 02-091 Poland; 4 Division of Veterinary Epidemiology and Economics, Institute of Veterinary Medicine, Warsaw University of Life Sciences-SGGW, Nowoursynowska 159c, Warsaw, 02-776 Poland; 5 Private Practice, Animal Veterinary Clinic, Młynarska 29, Łódź, Poland; 6 Department of Descriptive and Clinical Anatomy, Medical University of Warsaw, Chałubińskiego 5, Warsaw, 02-004 Poland; 7 Faculty of Materials Science and Engineering, Warsaw University of Technology, Wołoska 141, Warsaw, 02-507 Poland

**Keywords:** Hydrops fetalis, Transposition of great arteries, Aortic arch interruption, micro-CT, Fetal anasarca

## Abstract

**Background:**

*Hydrops fetalis* (HF) is fluid accumulation in fetus body cavities and subcutaneous tissue. The condition has been described in various farm and companion animal species, including dogs. Most of cases result from a heart defect. Exact nature of this defect is rarely clarified.

**Case presentation:**

A newborn, male French bulldog puppy with severe HF underwent a full anatomopathological examination to diagnose the primary cause of HF. Based on the anatomopathological examination, fetal ultrasound, and micro-computed tomography, transposition of the great arteries with hypoplasia of the ascending aorta, aortic arch interruption, ostium secundum atrial septal defect, severe tricuspid valve dysplasia, as well as hypoplasia of pulmonary vessels and lungs were diagnosed.

**Conclusions:**

This is the first report of HF caused by severe, complex congenital heart defects with concurrent pulmonary vessel and lung hypoplasia.

## Background

*Hydrops fetalis* (HF), also known as congenital edema or fetal anasarca, is not a diagnosis itself, but a syndrome that can accompany many diseases. The term refers to an excessive accumulation of fetal fluid in body cavities (abdominal and/or pleural cavity and/or pericardial sac) and subcutaneous edema. This congenital condition has been described both in the veterinary and human medical literature [1–3]. It has been reported in cows [4, 5], sheep [6, 7], goat [8], cats [9] and dogs [10]. In dogs, this syndrome is also referred to as swollen puppies, water puppies or walrus puppies. English and French Bulldogs, Pugs, Pekingese, and Boston Terriers are considered as predisposed breeds [10], however the syndrome has also been reported in a Briard-Beagle crossbred dog [11] and a flat-coated Retriever [12]. HF may develop in one or more fetuses in the litter and can cause obstructive dystocia [10]. Most of affected puppies die, usually shortly after birth. In human medicine, HF may be either immune-mediated or non-immune syndrome. The former had been mostly caused by *Rhesus* isoimmunization, however, effective prophylaxis led to a substantial decrease in its incidence. Currently, it is estimated that non-immune HF accounts for up to 90% of all reported cases [13]. Recent studies on human fetuses with HF have revealed the following causes: heart disease (15.2%) including cardiomyopathy, Ebstein’s anomaly, pulmonary valve stenosis, coarctation of aorta, aortic valvular stenosis, hypoplastic left heart, atrioventricular canal defect syndrome, mitral valve insufficiency, pulmonary artery anomaly, pulmonary artery atresia, double outlet right ventricle, single ventricle, myocarditis, tetralogy of Fallot, truncus arteriosus, coronary artery fistula, hypoplastic right heart, total anomalous pulmonary venous return, tricuspid valve atresia, aortic malformations, interrupted aortic arch, transposition of the great vessels (TGA), genetic causes (12.5%), arrhythmias (8.2%), infectious diseases of the fetus (7.6%), congenital anemia (5.2%), hemolytic anemia (3.8%), and twin-to-twin transfusion syndrome (3.8%). In nearly 30% of cases, the cause remains unknown [3, 14]. HF in humans may also be caused by heart tumors of the fetus. In the presence of pathological masses in the heart, HF occurs due to impaired blood flow. The most commonly diagnosed heart tumor is rhabdomyoma and much less common fibroma, teratoma, and hemangioma [15–17]. Based on very limited data from veterinary medicine cardiac malformations appear to be the most common cause of HF, but little data is available in animals [11]. Both in humans and animals, HF can be diagnosed in the prenatal ultrasound [10, 18].

## Case presentation

**Fig. 1:**
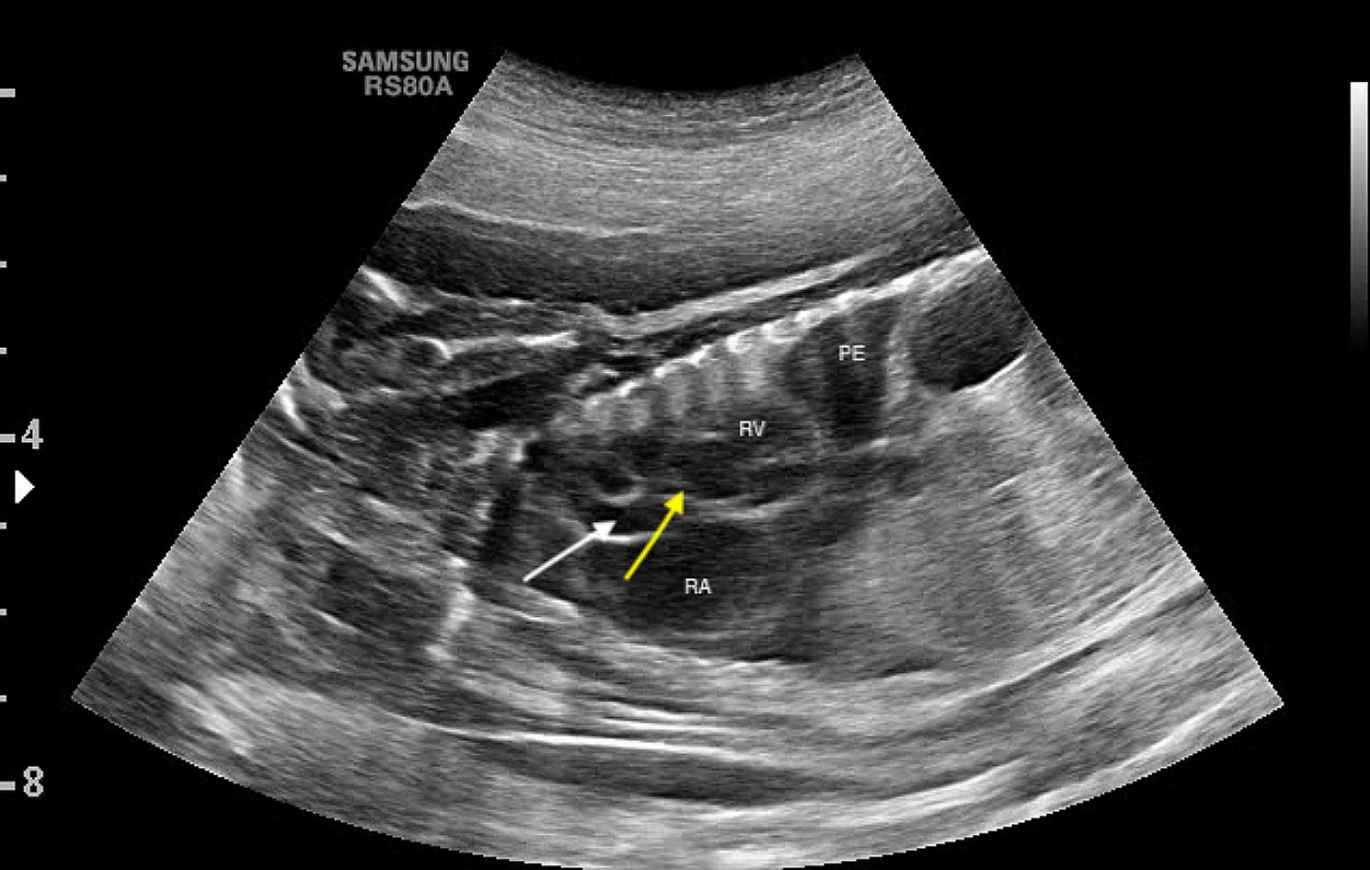
Fetal ultrasound scan of the puppy with hydrops fetalis. The yellow arrow points to a hypoplastic artery coming from the right ventricle, the white arrow points to a properly developed artery coming from the left ventricle. PE – pleural effusion, RA – right atrium, RV – right ventricle

**Fig. 2:**
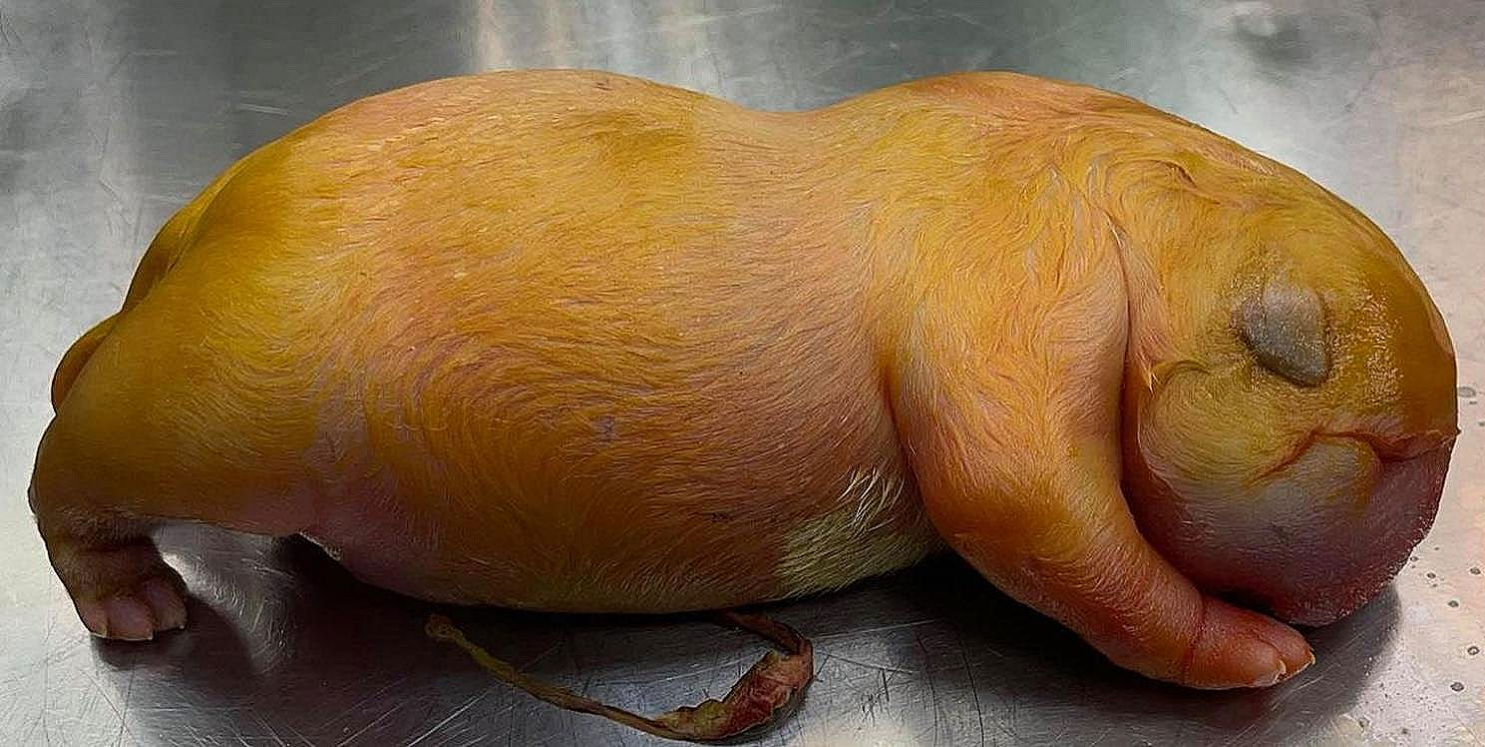
The generalized subcutaneous tissue swelling in puppy with hydrops fetalis

**Fig. 3:**
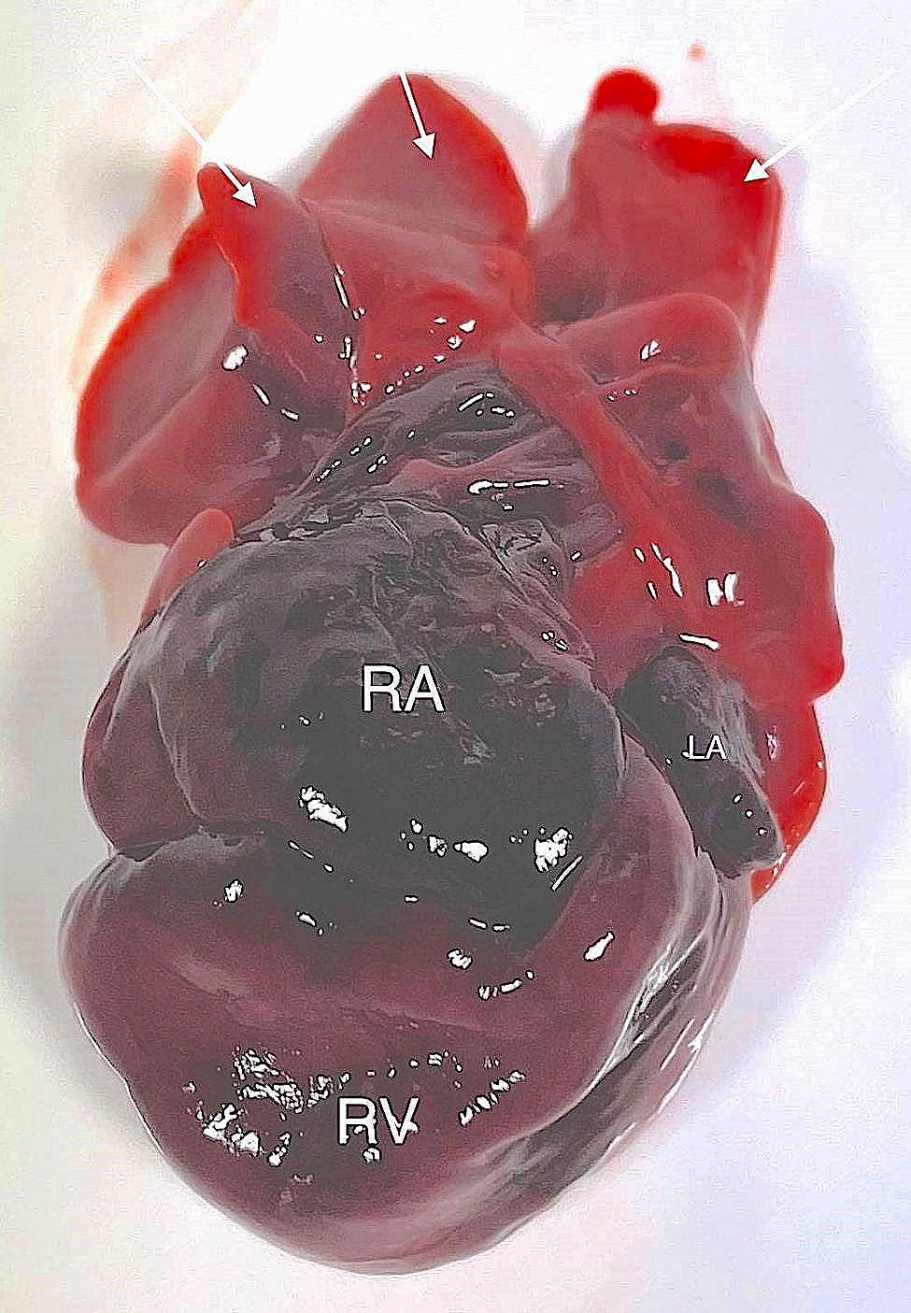
The heart and lungs of the puppy with hydrops fetalis. White arrows point to hypoplastic lung lobes. RA – right atrium, RV – right ventricle, LA – left atrium

**Fig. 4:**
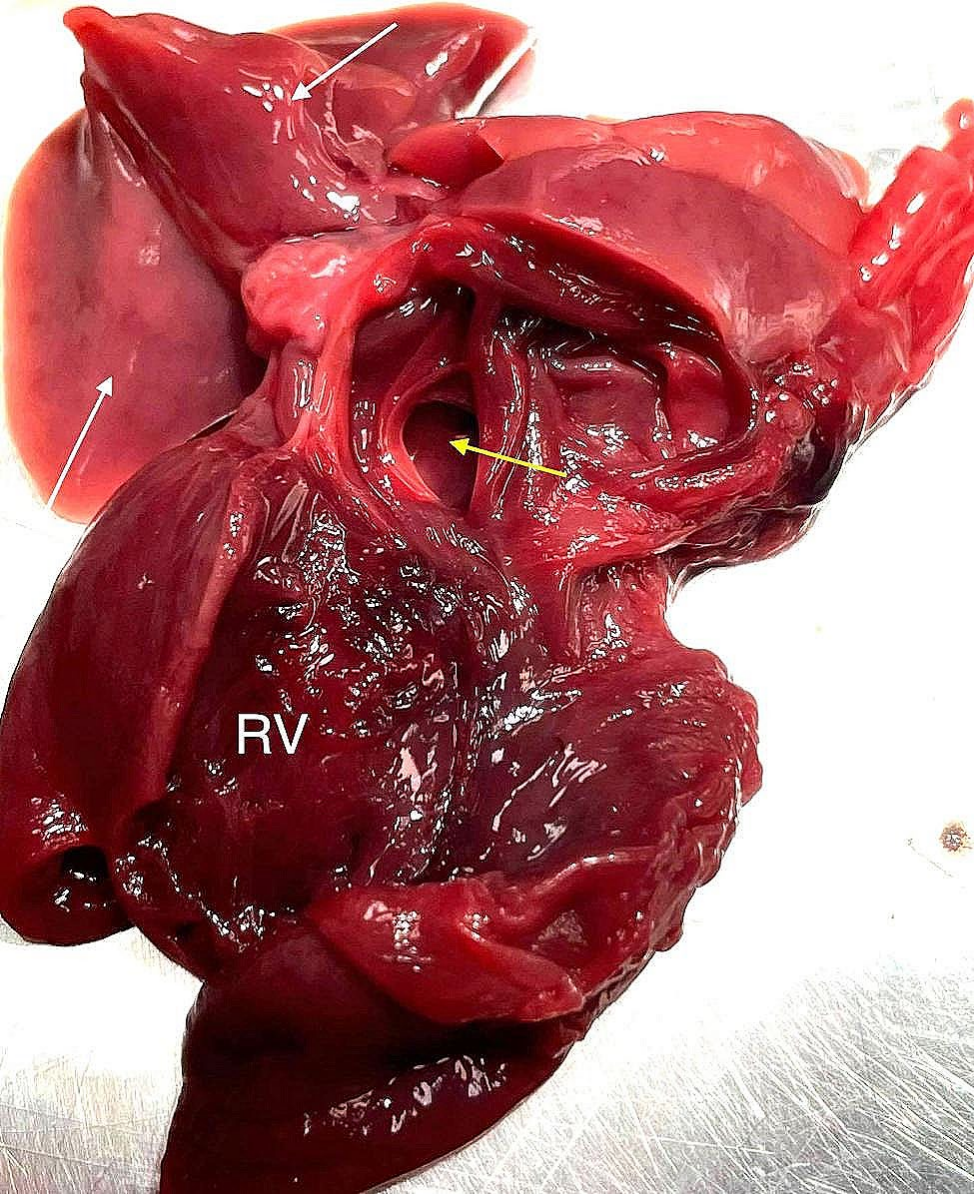
The heart of the puppy with hydrops fetalis revealing hypoplastic lung lobes (white arrows) and the atrial septal defect (yellow arrow). RV – right ventricle

**Fig. 5:**
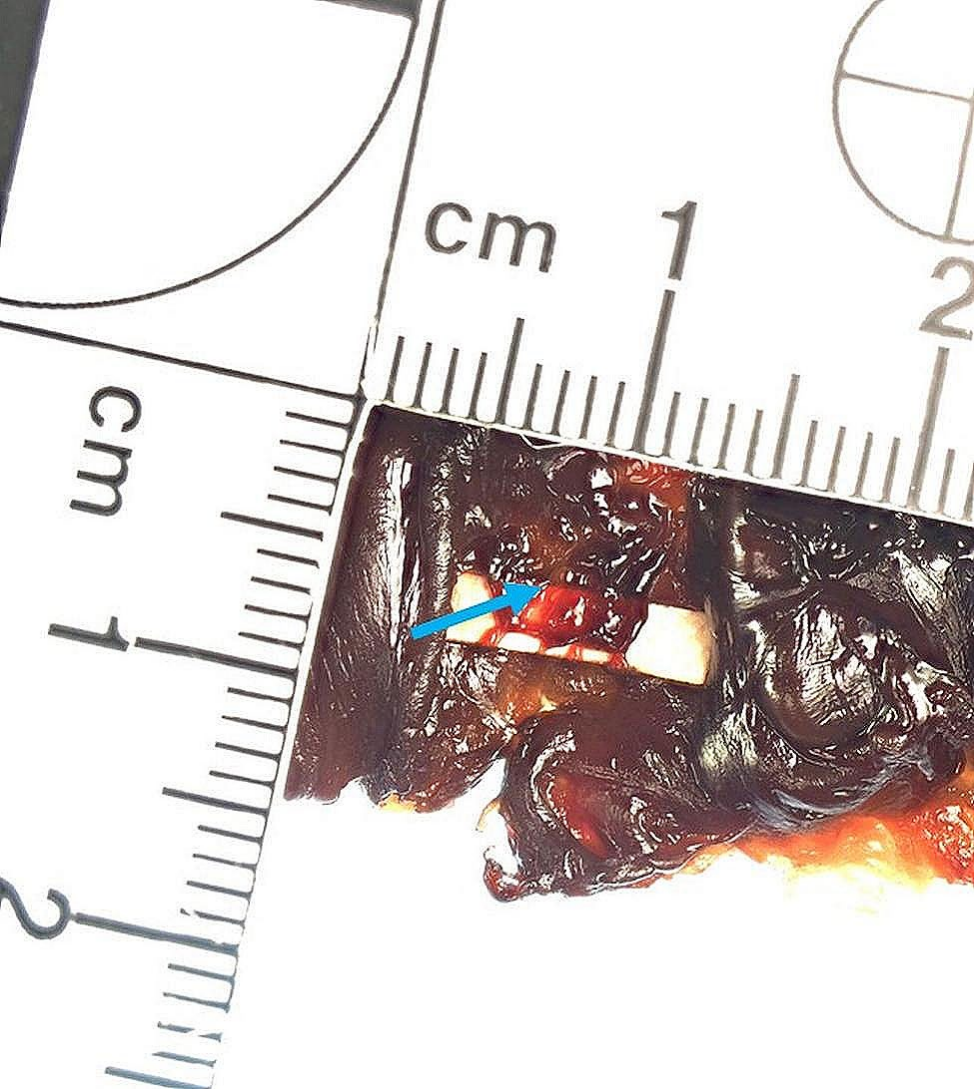
The heart of the puppy with hydrops fetalis revealing the septal leaflet of the tricuspid valve (blue arrow). A white indicator placed under the septal valve leaflet visualizes an incorrect attachment of very short chordae tendinea directly to the septum

A 3 year-old pregnant female French Bulldog was presented to the veterinary clinic in the 58th day of pregnancy as calculated based on the date of mating. The female was in the second pregnancy whose course had so far been uneventful and she had not received any medications or supplements during pregnancy. The abdominal ultrasound confirmed the advanced pregnancy with the presence of a single fetus located in left uterine horn. No pathologies were found in the abdominal cavity of the female. The fetal ultrasound revealed an accumulation of fluid in the thoracic and abdominal cavities as well as subcutaneous tissue, mainly around the neck and head and a severely enlarged right atrium and right ventricle of the heart. The great arteries were visibly located parallel to each other, one clearly hypoplastic with a diameter of about 1 mm, and the other with a diameter of 4 mm (Fig. 1). A caesarean section was performed immediately, and one alive puppy with severe HF was obtained (Fig. 2). The male puppy, weighing 238 g, was lethargic and died within an hour. Due to the diagnosed congenital heart defect identified during prenatal ultrasound, resuscitation was not performed. A full anatomopathological examination was performed. It revealed generalized swelling of the subcutaneous tissue, a large amount of free fluid in the thoracic and abdominal cavity and a small amount of fluid in the pericardial sac, lung hypoplasia and massive enlargement of the right atrium and right ventricle of the heart (Fig. 3). The liver and kidneys were also enlarged. A detailed examination of the heart was performed according to the protocol for congenital heart defects [19, 20]. First, the pericardial sac was gently removed. One great artery coming out of the heart was visible without connection to the lungs and the vascularization of the lungs was abnormal. The first heart incision was made along the long axis of the right atrium to reveal the interatrial septum and to assess the patency of the foramen ovale. A large atrial septal defect was visualized (Fig. 4). Then, the right ventricle was cut along the interventricular septum. After its full opening, the dysplastic, thickened tricuspid valve was uncovered. Its septal leaflet had very short chordae tendinea attached to the interventricular septum which significantly reduced its mobility (Fig. 5). Subsequently, the left part of the heart was examined, starting from incision along the left atrium and incision made along the left ventricle at the interventricular septum to visualize the normally developed mitral valve.

**Fig. 6:**
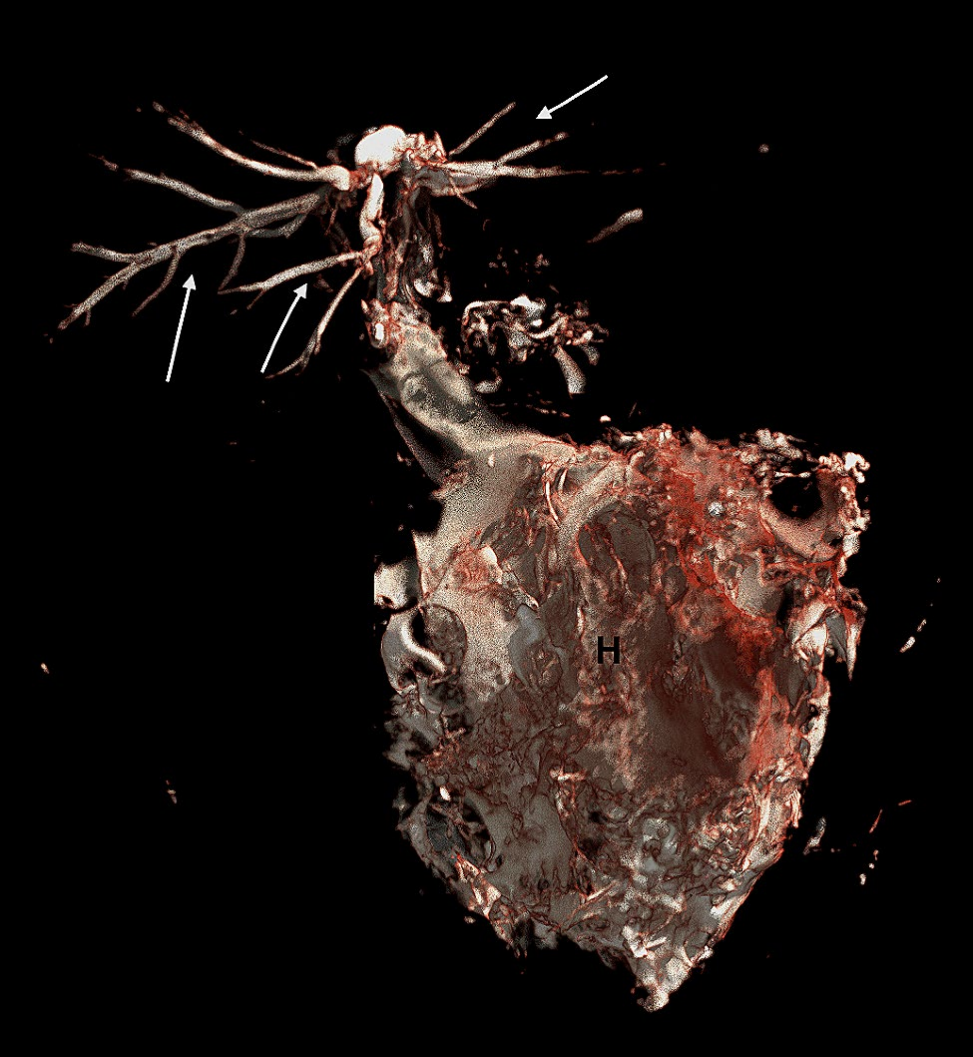
Micro-CT with contrast medium scan of the heart of the puppy with hydrops fetalis revealing hypoplastic pulmonary vessels (white arrows). H – heart

**Fig. 7:**
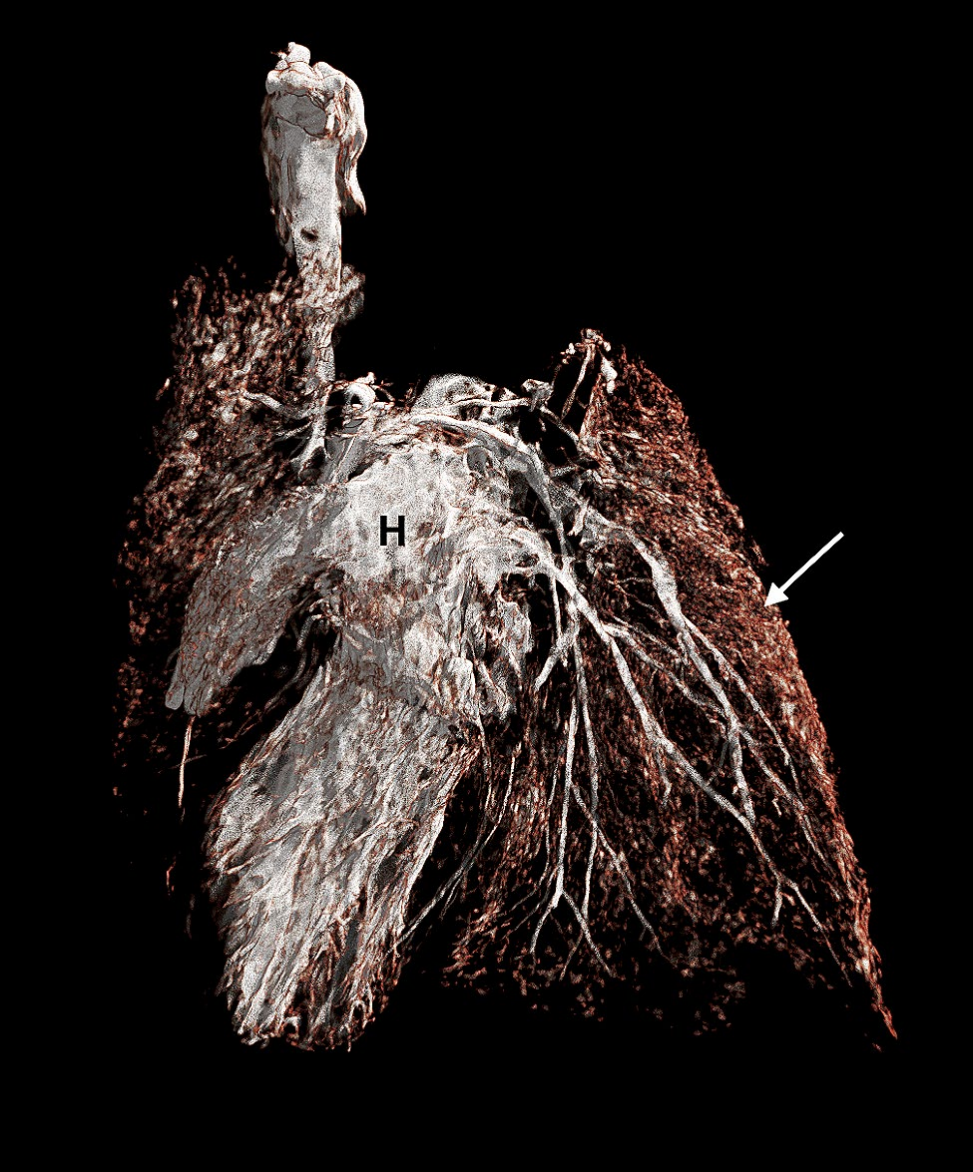
Micro-CT with contrast medium scan from a different puppy which died immediately after birth with properly developed lungs. The white arrow points to properly developed vascularization of the lungs. H – heart

**Fig. 8:**
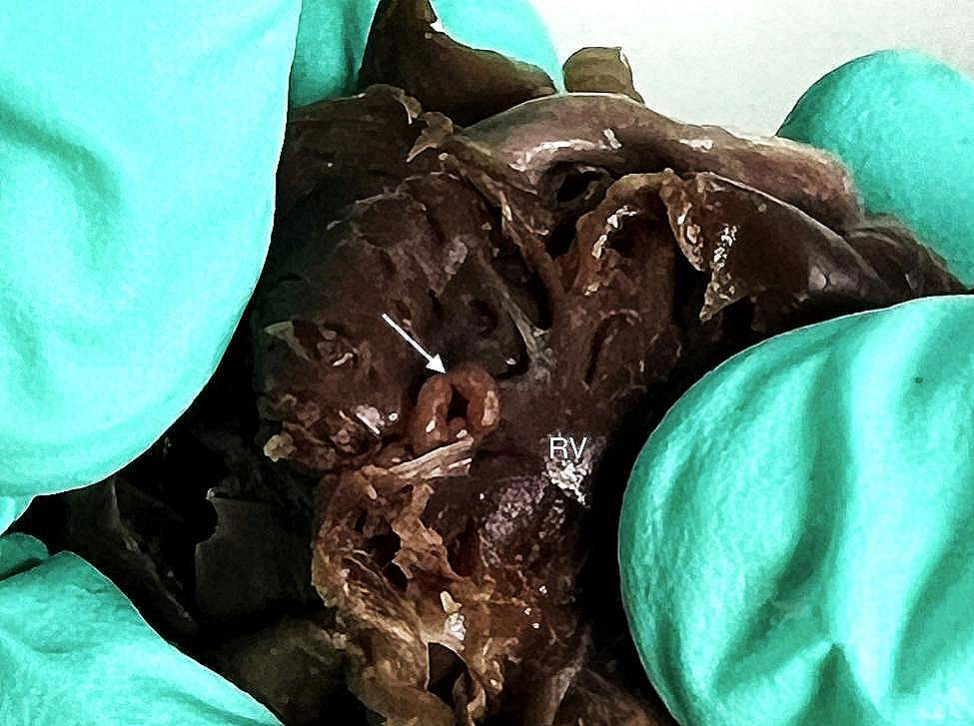
The heart of the puppy with hydrops fetalis revealing the hypoplastic aortic orifice emerging from the right ventricle (white arrow) and with lumen narrowed by the thickening of the surrounding tissues. RV – right ventricle

**Fig. 9:**
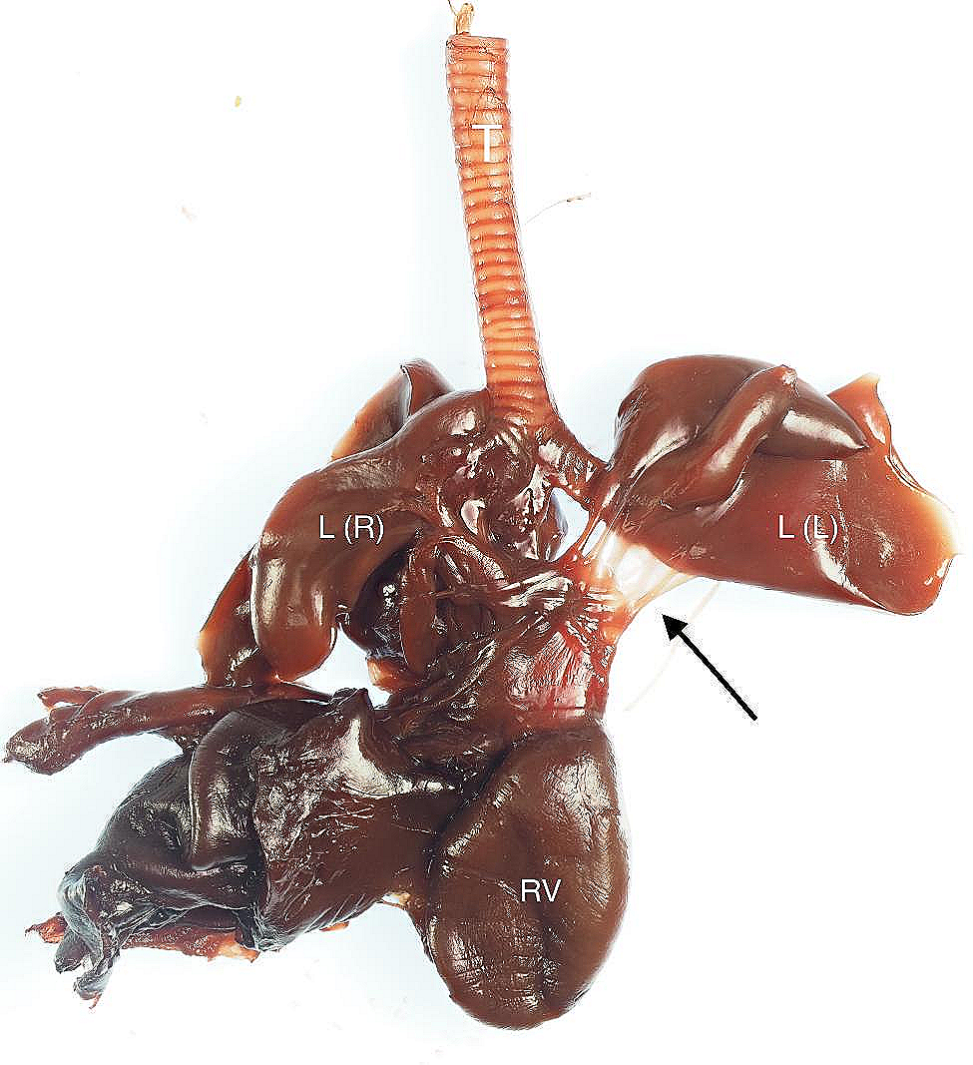
The heart, lungs and respiratory tract excised from the puppy with hydrops fetalis. T – trachea, L (L) – left lung lobes, L (R) – right lung lobes, black arrow point to hypoplastic vascularization of the lungs, no clear origin of the left pulmonary artery proximal part is visible

**Fig. 10:**
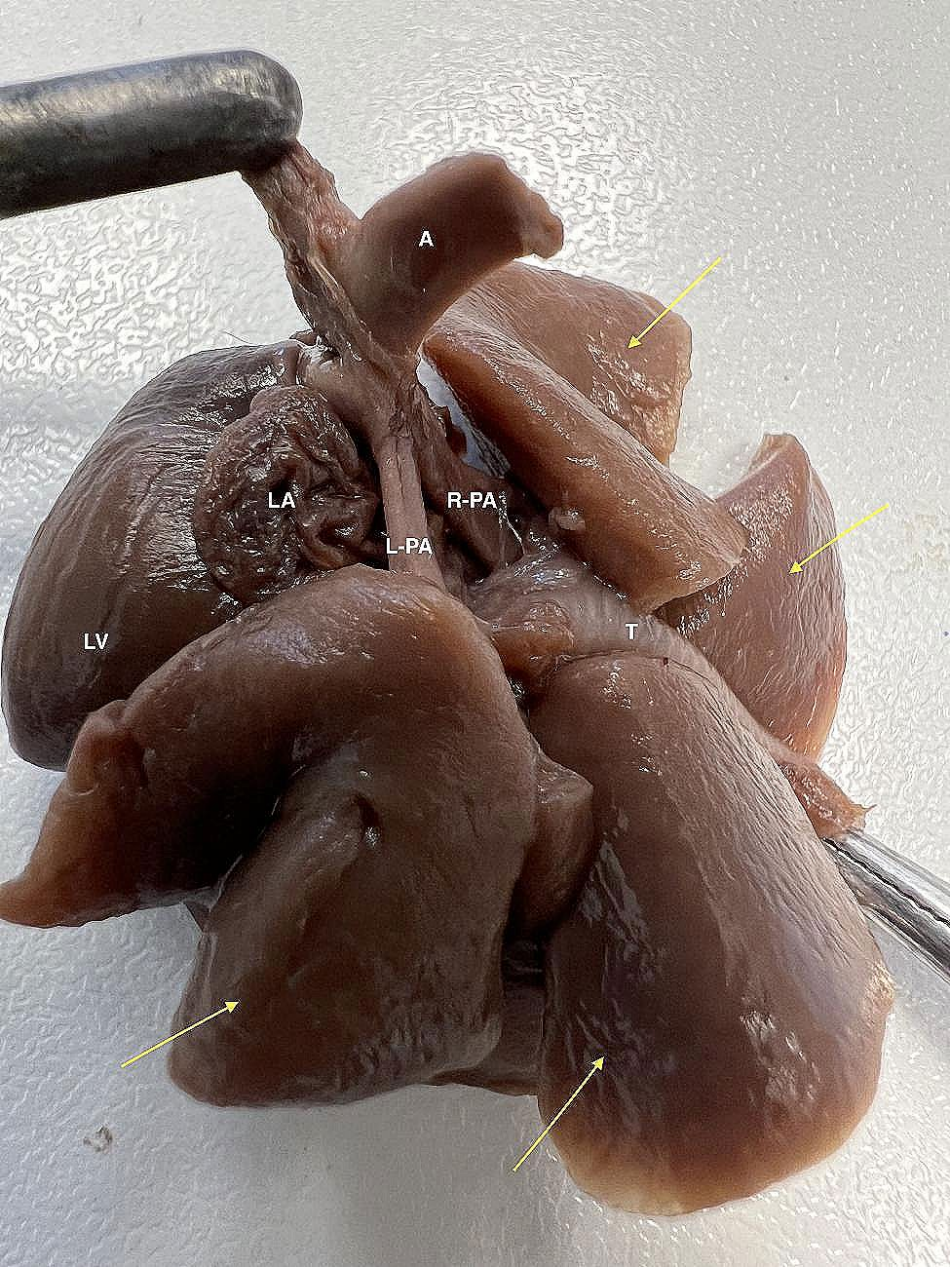
Post-mortem picture of a puppy with properly developed lungs and the right and left pulmonary arteries correctly branching from the pulmonary trunk. LA – left atrium, LV – left ventricle, A – aorta, L-PA – left pulmonary artery, R-PA – right pulmonary artery, T – trachea, yellow arrows point to the lung lobes

Due to abnormal morphology of the heart and coexisting hypoplastic lungs a micro computed tomography (micro-CT) examination was performed before further incisions. Surgical sutures were placed on all incisions. Then, the cranial and caudal vena cava were ligated. Also the brachiocephalic trunk and left subclavian artery were ligated and a Foley catheter (8Fr/Ch, ZARYS International Group, Poland) was placed into descending aorta. Then, the balloon was slowly inflated with water from the attached 5 ml syringe, so that the catheter tightly filled the lumen of the artery. Contrast medium containing a mixture of 8 g of pork gelatin dissolved in 50 ml of hot water (temperature 95ºC) and 25 ml of barium sulfate (Barium sulfuricum Medana 1 g/ml, Polpharma, Poland) was administered through the catheter until the heart along with the visible parts of coronary arteries was fully filled. After removal of the catheter, the aorta was ligated. Then, the heart and lungs were placed in a 100 ml plastic container. The container was filled with a mixture of 8 g agar (Sigma-Aldrich, USA) dissolved in 100 ml of water in a water bath. Organs were completely covered by the mixture. Embedding in agar immobilized organs during micro-CT examination. The organs were not fixed in formalin to avoid stiffening of the tissues, which hampers subsequent anatomopathological examination. This was especially important given a very small size of the examined organs – the approximate diameter of the examined heart was less than 2 cm. A micro-CT examination was performed using Xradia XCT-400 (Carl Zeiss Microscopy, Germany), to visualize the pulmonary vessels and to determine the cause of lung hypoplasia. The post-processing analysis was performed using CTVox volume rendering, DATAVIEWER 64 bit version (Bruker, Belgium) and Horos v2.4 software. The micro-CT images were reviewed by a human medicine radiologist experienced in heart imaging (WM) and a veterinary cardiologist (OSJ). After the examination, the heart chambers were cleared of contrast and the anatomopathological examination was continued. The micro-CT examination showed hypoplastic vascularization of the lungs (Fig. 6). A micro-CT scan from a different puppy that died immediately after birth with properly developed lungs and right and left pulmonary arteries properly branching from the pulmonary trunk is presented for comparison (Fig. 7). The next incision was made towards the right ventricle outflow tract to visualize the artery valve. Then, the incision was extended to the entire length of the artery. The last incision was made towards the left ventricle outflow tract to visualize the artery valve and was extended to the entire length of the artery. The outflow tract from the right ventricle ended in a hypoplastic ostium (Fig. 8). There were orifices of the coronary vessels in the hypoplastic artery coming out of the right ventricle. The vessel itself, after leaving the heart, branched into the brachiocephalic trunk, and left subclavian artery without any further connection with the descending aorta. The pulmonary trunk with properly formed valve emerged from the left ventricle and joined the descending aorta through the ductus arteriosus. Abnormally developed, hypoplastic left pulmonary veins and arteries were visible, without possibility to determine the exact point of departure from the heart (Fig. 9). The anatomy of the right lung vascularization either could not be precisely revealed. A picture from an autopsy of a different puppy that died immediately after birth with properly developed lungs and right and left pulmonary arteries properly branching from the pulmonary trunk is presented for comparison (Fig. 10). The vascularization of both lungs was clearly hypoplastic and the lungs were considerably smaller than normal. Also, the ratio of the lung vascularization to the heart size visible in the micro-CT examination was different. After all procedures, the heart was placed in a 4% buffered formaldehyde solution (approx. 10% Formalin solution dissolved in water solution of (di)sodium (di)hydrogen orthophosphate).

## Discussion and conclusions

In this case report, we describe a newborn puppy with complex heart defect consisting of the TGA with hypoplasia of ascending aorta, aortic arch interruption, ostium secundum atrial septal defect and severe tricuspid valve dysplasia. As far as we know, this is the first case of HF with pulmonary hypoplasia caused by a complex congenital heart defect (CHD) in the dog. Such a detailed diagnosis could be established only thanks to a combination of fetal ultrasonography, autopsy, and micro-CT examination. Although we tried very hard, we could not localize the proximal sections of the pulmonary vascularization. It is most likely that the vascularization originated from the collaterals of the ductus arteriosus or aorta, especially since the pulmonary vessels filled immediately after the contrast was administered to the descending aorta. Finally, a complex heart defect, accompanied by hypoplastic, abnormal pulmonary vascularization, and a secondary lung hypoplasia were diagnosed.

Knowledge about lethal complex CHDs in dogs is limited. In French Bulldogs, the most commonly described CHD is pulmonary artery stenosis [21]. Pedigree analysis in English and French Bulldogs affected by subvalvular aortic stenosis (SAS) suggests an autosomal recessive pattern of inheritance [22]. This represents a different inheritance pattern compared to SAS in Newfoundlands, where it is believed to be transmitted via an autosomal dominant pattern and linked to a single genetic mutation [23]. Still many aspects of the genetic causes of other CHDs in these breeds warrant clarification. Complex CHDs may be the cause of HF but detailed data in animals is lacking. In humans, it is estimated that HF resulting from CHD occurs in 20% of cases [20] The situation is similar to the pulmonary hypoplasia in dogs. Only a few cases have been reported, but most affected animals survived at least several months after birth [24, 25]. Lung morphogenesis is a complex process, and the development of pulmonary vascularization is believed to have a strong influence on the development of the lung tissue. Early pulmonary vascular disorders impair the morphogenesis of airway branches and interfere with lung tissue formation. A reduced pulmonary blood flow results in a high risk of pulmonary hypoplasia [26]. It seems highly probable that the pulmonary vascular hypoplasia in the described case also resulted from a severe, complex CHD and abnormal blood supply to the pulmonary vessels. Survival rate in children born with HF depends on the primary cause and is estimated at 27–48% [27]. No such data are available for animals. It is known from human medicine that only the surgical correction may significantly improve prognosis in severe CHDs. In infants such operations give a high chance of long-term survival – the 5-year survival rate after aortic arch surgery is estimated at 86% [28]. After the surgical correction of the TGA a 20-year survival rate exceeds 75% [29]. However, in veterinary medicine surgical treatment of such complex CHDs is not feasible.

## Data Availability

Not applicable.

## Abbreviations

CHD: Congenital heart defect
HF: Hydrops fetalis
micro-CT: Micro–computed tomography
SAS: Subvalvular aortic stenosis
TGA: Transposition of great arteries
